# The Double Layer Methodology and the Validation of Eigenbehavior Techniques Applied to Lifestyle Modeling

**DOI:** 10.1155/2017/4593956

**Published:** 2017-01-04

**Authors:** Giuseppina Schiavone, Bishal Lamichhane, Chris Van Hoof

**Affiliations:** Wearable Health Solutions, Holst Centre, High Tech Campus 31, 5656 AE Eindhoven, Netherlands

## Abstract

A novel methodology, the double layer methodology (DLM), for modeling an individual's lifestyle and its relationships with health indicators is presented. The DLM is applied to model behavioral routines emerging from self-reports of daily diet and activities, annotated by 21 healthy subjects over 2 weeks. Unsupervised clustering on the first layer of the DLM separated our population into two groups. Using eigendecomposition techniques on the second layer of the DLM, we could find activity and diet routines, predict behaviors in a portion of the day (with an accuracy of 88% for diet and 66% for activity), determine between day and between individual similarities, and detect individual's belonging to a group based on behavior (with an accuracy up to 64%). We found that clustering based on health indicators was mapped back into activity behaviors, but not into diet behaviors. In addition, we showed the limitations of eigendecomposition for lifestyle applications, in particular when applied to noisy and sparse behavioral data such as dietary information. Finally, we proposed the use of the DLM for supporting adaptive and personalized recommender systems for stimulating behavior change.

## 1. Introduction

Managing health requires a holistic understanding of the individual's behavior [1]. This is particularly the case if one wants to predict the probability of occurrence of a disease or assess the quality of life after treatment or track relapses of certain addictive behaviors, such as smoking, alcohol, and drug abuse. The ability to recognize, model, and track behaviors is paramount to promote self-awareness and should be a fundamental feature of any personalized recommender system aiming for behavioral change. Over the past decade, most attention has been directed towards recognition problems, such as activity recognition [2] or emotion detection [3]. Personalized temporal modeling of human behavioral patterns remains largely unexplored [4]. Daily diaries and surveys are still the most extensively adopted methods in longitudinal research studies and in medical practice to grasp qualitative insights into a person's routines. The implementation of diary annotation tools in self-tracking mobile apps, such as MyFitnessPal, Endomondo, Momentum, and HabitBull, has made self-reporting less cumbersome and more systematic, also allowing the users to acknowledge their progress during the attainment of personal goals. Nevertheless, truly automated analysis of e-diaries and self-reports for modeling individuals' behavior dynamics is not yet available.

In this work, we propose the use of machine learning and eigendecomposition techniques to detect individuals' routines captured from self-reporting of daily diet and daily activities and to find behavioral correlates of health indicators. The main objectives of this work are to

- test the feasibility of the eigenbehavior technique (initially presented by Eagle and Pentland in 2009 [5] to model social network dynamics) for lifestyle tracking applications,
- propose a novel approach, referred here to as* double layer methodology (DLM)*, to model the relationship between individuals' health indicators and their behavior,
- discuss the implications of our approach for the design of adaptive and personalized recommender systems.

After describing the study cohort (in Section 2.1), the knowledge representation method and the generation of behavioral matrices are presented (in Sections 2.2 and 2.3, resp.). In Section 2.4, we provide a description of the eigenbehavior technique, following similar notation as in Eagle and Pentland [5]. Next (in Section 2.5) we outline the main features of our implementation, explaining the* double layer methodology*. In* Results* (1) we present the application of unsupervised clustering method to group individuals on the basis of health indicators; (2) we show the primary principal components of diet and activity behaviors at both individual and group level; (3) we demonstrate the ability to predict the subsequent dietary and activity behaviors within a day; (4-5-6) we illustrate the use of Euclidean distance to uncover within-individual similarities across days, to detect differences between individual pairs and to determine an individual's belonging to a particular group; (7) and, finally, we report the evaluation of health indicators-based clustering. Afterwards we discuss related work and conclude by proposing possible extensions of the* DLM*.

## 2. Material and Methods

### 2.1. Cohort Selection

The subjects in this study were part of a bigger study of 75 subjects [6], which had the objective of developing calibration-free algorithms for fitness level estimation of healthy individuals in free living conditions. All the subjects were recruited within the university environment. Inclusion criteria were no report of medical conditions and body mass index (BMI) in the range 18.5–27 kg/m^2^. Exclusion criteria were age below 18 and above 45, smokers, presence of chronic diseases, and presence of musculoskeletal conditions preventing exercise. The subjects were observed for a maximum period of 2 weeks. Observations consisted of (1) a series of laboratory tests (submaximal VO2max test and exercises at different intensities) performed over two days of the observation period, at the beginning and in the middle of the 2 week period; (2) recordings in free living conditions, consisting of continuous monitoring of a subject's activity and physiology using a wearable device. During this period, the subjects were asked to fill out daily diaries and report their activity and diet. For all subjects, anthropometrics (such as weight and height) and fitness level (VO2max) were recorded. The study was approved by the university ethics committee and signed informed consent was obtained from all of the participants.

In this work, diary data and anthropometrics from only 21 of the subjects (gender: 8 males, 13 females) have been used because the other subjects did not provide sufficient annotation about their diet. The mean number of annotated days per subject was 10. A summary of the study population demographics and additional readings, such as VO2max, relative VO2max, fat mass, fat free mass, percentage of fat mass, rest metabolic rate (RMR), and basal energy expenditure (BEE), are reported in Table 1.

### 2.2. Data Extraction and Knowledge Representation

Daily activity and diet were manually annotated by the subjects in a table format, where the start and end time of the activity and time of food item consumption were also indicated. Preprocessing techniques, such as tokenization, word removal, spell checking, and lemmatization, were applied for the analysis of the provided annotations. Additionally, words were separated into two categories, one for diet and the other for activity and grouped into classes as shown in Table 2.

### 2.3. Behavior Matrices

After preprocessing, the activity and diet data were treated separately representing two behavioral spaces. Temporal information was included by considering activity and diet annotation in different periods of the day. For activity classes, each day was divided into three periods:

- morning (P0, from 00:00 to 12:00),
- afternoon (P1, from 12:00 to 17:00),
- evening (P2, from 17:00 to 24:00).

For diet classes, each day was divided into six periods:

- breakfast (P0, from 00:00 to 09:00),
- morning (P1, from 09:00 to 12:00),
- lunch (P2, from 12:00 to 14:00),
- afternoon (P3, from 14:00 to 17:00),
- dinner (P4, from 17:00 to 19:00),
- evening (P5, from 19:00 to 24:00).

Successively, binary behavior matrices, *B*(*x*, *y*), were generated for each subject. Each row of *B*(*x*, *y*) corresponds to an individual's behavior, Γ_*i*_, over a day *i*, and each column corresponds to a class at a given period of the day, where 1 indicates the presence of a class. For example, sport_P0 is set to 1, if the subject annotates a sport activity in the morning. The absence of the class was indicated by 0 (e.g., set caffeine drink_P5 to 0 if the subject did not drink coffee in the evening). The behavior matrices have dimensions* D by H*.* D* represents the number of days of diary reported by a subject.* H* has a dimension of* n by m*, with *n* indicating the number of periods in the day and *m* indicating the total number of classes per category. Individual behavior matrices were used as the feature space for the analysis at individual level. Behavior matrices for analysis at group level were also generated; in this case each row of a matrix represents the average behavior of an individual belonging to the group.

**Figure 1:**
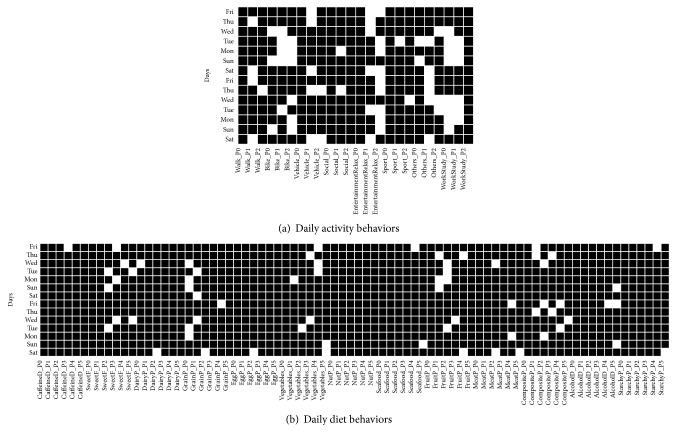
Binary behavior matrices for one subject who annotated a 14-day diary. (a) The activity behavior matrix, each column corresponding to the activity classes as in Table 2, separated into 3 daily periods; (b) the diet behavior matrix, each column corresponding to the diet classes as in Table 2, separated into 6 daily periods. Each row corresponds to a daily behavior; a white square corresponds to a performed activity or consumed items.

The behavior matrix for one subject with annotations provided for 14 days is shown in Figure 1. This particular subject reported the use of an automated vehicle on Saturdays and Thursdays during the day (class name:* vehicle_P1*) and he/she generally reported relaxation and/or entertainment activities in the evening (class name:* entertainmentRelax_P2*). The annotation information about the diet is quite sparse.

### 2.4. Eigen-Behavior Analysis

Eigenbehavior analysis was proposed by Eagle and Pentland (2009) [5] to identify and predict individual and community behaviors within a social network using mobile phone information [7]. The underlying hypothesis of this approach is that there is a set of repetitive behaviors or routines that can be recognized at an individual and group level. This routine can be used to predict daily behavior and determine similarities between individuals and their affiliation to a community. Eigendecomposition is used to identify such routines. Primary eigenbehaviors are defined as the principal components of routines and correspond to the eigenvectors,* u*, of the covariance matrix of the behavior data, as defined in Section 2.3, with the largest eigenvalues, *λ*. Each eigenbehavior represents a vector of *M* values, each associated with a class of the behavior space (diet or activity space in our case). Individuals for which a smaller number of primary eigenbehaviors is able to explain up to 90% of the behavior variability can be said to exhibit a more regular behavior compared to individuals having a higher number of primary eigenbehaviors. The linear combination of an individual's primary eigenbehaviors can be used to reconstruct the behavior of each day in the data. Additionally, Eagle and Pentland (2009) [5] showed that when primary eigenbehaviors are calculated for an individual, it is possible to infer the projection of an entire day using information from only a portion of that day. We will show how such an approach was also applied to our data. The average behavior of each individual, Ψ = (1/*D*)∑_*n*=1_^*D*^Γ_*n*_, (with *D* total number of days per subject), can be used to define the behavior space of the community to which the individual belongs. In this case, the behavior matrix has dimension *N*, number of individuals in the community, by *H*. Eigendecomposition can then be applied again to the community behavior data to determine the primary eigenbehaviors of the community, *u*_*k*_^*j*^ (*k* is the number of primary eigenbehaviors for the community* j*). Projection of the individual behaviors into the community behavior space is used to determine the distance between individuals belonging to a community from the others. Such projections are obtained by calculating the vector of weights, *Ω*^*j*^ = [*ω*_1_^*j*^, *ω*_2_^*j*^, *ω*_3_^*j*^,…, *ω*_*M*_^*j*^], representing the optimal weighting configuration to get an individual's behavior as close as possible to the community behavior space, with weights being defined as *ω*_*k*_^*j*^ = *u*_*k*_^*j*^(Γ − Ψ^*j*^) (with Γ the individual's behavior or individual's average behavior and Ψ^*j*^ the average behavior of the community* j*). Euclidean distance between weight vectors of an individual, *Ω*^*j*^, and the others, *Ω*_*l*_^*j*^, projected in the same community behavioral space,* j*, and defined as *ε*_*jl*_^2^ = ‖*Ω*^*j*^ − *Ω*_*l*_^*j*^‖^2^, is used as a measure of similarity between individuals [8]. Similarly, similarities between days for an individual can be obtained from *ε*_*ld*_^2^ = ‖*Ω*^*l*^ − *Ω*_*d*_^*l*^‖^2^, where the weight vector for one day *Ω*^*l*^ is defined as *Ω*^*l*^ = [*ω*_1_^*l*^, *ω*_2_^*l*^, *ω*_3_^*l*^,…, *ω*_*M*_^*l*^], and *Ω*_*d*_^*l*^ corresponds to the weight vectors for the other days,* d*, and where the weights for each subject are defined as *ω*_*k*_^*l*^ = *u*_*k*_^*l*^(Γ^*d*^ − Ψ^*l*^), with Γ^*d*^ representing the individual's daily behavior and Ψ^*l*^ = Γ is the individual's average behavior. Euclidean distance can also be applied to determine the similarity of an individual behavior to the community behavior as a whole and thus as a tool to estimate proximity or affiliation of an individual to a community. In this case, such distance is defined as follows: *ε*_*j*_^2^ = ‖*ϕ*^*j*^ − *ϕ*_*b*_^*j*^‖^2^, where *ϕ*^*j*^ = Γ − Ψ^*j*^ represents the mean-adjusted behavior of the individual and *ϕ*_*b*_^*j*^ = ∑_*i*=1_^*M*^*ω*_*i*_^*j*^*u*_*i*_^*j*^ is its projection onto the community's behavior space.

### 2.5. From Social Network Analysis to Lifestyle Pattern Recognition

Recognizing dietary and activity behavioral patterns across an individual's lifespan and identifying collective behaviors, such as the lifestyle of fitness enthusiasts or the lifestyle of sedentary people, is somewhat different than defining behavioral dynamics of individuals and communities in a social network. In particular, meanwhile it is trivial to make community distinctions, for example, to cluster students on the basis of their belonging to one or another school; in the case of lifestyle, considered as a correlate of health, the definition of grouping is an ethically delicate and ambiguous problem. This is because in real-life scenarios, people are generally reluctant to be clustered and can often exhibit behaviors that are common to several groups; for example, not all the people that eat sweets are obese. Additionally, the definition of health is generally not binary; for example, even within a population of people with chronic diseases, some will be healthier than others according to their physical and mental ability to adapt and self-manage [9].

**Figure 2:**
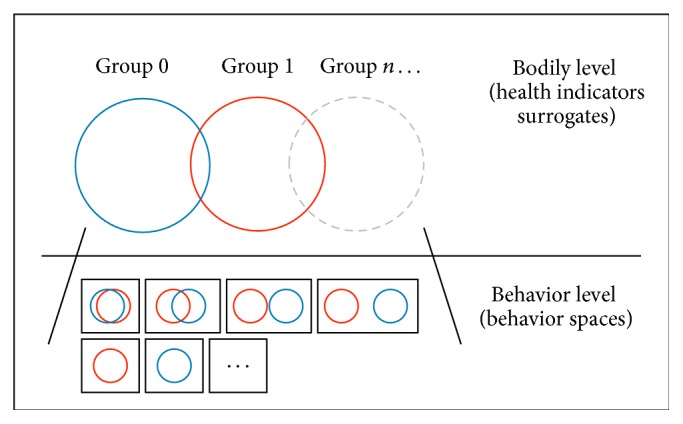
Illustration of the proposed* double layer methodology* consisting of (i) grouping individuals on the basis of their health indicators (as in Table 1) and (ii) identifying emerging individual behavioral dynamics. Individuals belonging to a group can exhibit a diverse range of behaviors from behavior dynamic typical of his/her group to behavior dynamics proper of other groups.

To overcome this problem, we propose the* double layer methodology* (*DLM*). The DLM is a pragmatic approach for grouping individuals of a population, which does not rely on the individual's behavior, but on the individual's physical characteristics, such as the factors listed in Table 1. The reasoning behind this choice is that bodily, physiological, and metabolic characteristics, such as weight, fitness level [10], and resting metabolic rate [11], here referred to as health indicators, have an impact on lifestyle and on dietary and activity preferences. They can partially contribute to the exhibition of specific behaviors and can provide indications of the individual's health condition. The DLM is then composed of two layers. On the first layer or bodily level, individuals are grouped on the basis of health indicators. On the second layer, individuals' and groups' behaviors are evaluated and compared. A schematic representation of the DLM is shown in Figure 2.

**Figure 3:**
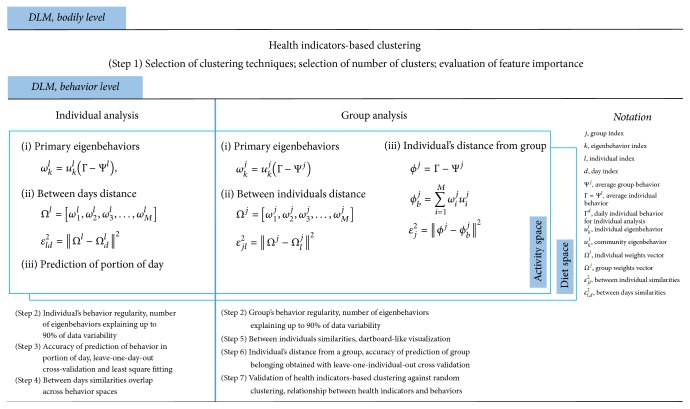
Summary of the steps in the DLM. At each level of the DLM (bodily and behavior), the different steps are numbered as explained in the main text. Steps adopted per individual and group analysis are separated by the thick blue line.

The DLM, presented in this work, was implemented in python and organized in the following steps (also summarized in Figure 3).

*(1) Clustering of Population by Bodily, Physiological, and Metabolic Characteristics*. At the bodily layer of DLM, we compared two different unsupervised clustering techniques,* k*-means and spectral clustering, to uncover the number of groups in our population on the basis of individuals' health indicators.* K*-means (settings: defaults from the scikit library implementation in python) and spectral clustering (settings: affinity matrix obtained with cosine similarity) were investigated because of their suitability for a small dataset. Hierarchical clustering methods and grid-based methods were not considered for this analysis because they are more suitable for larger datasets [8]. After selecting the final clustering technique and the optimal number of clusters, using Silhouette score as performance metric, we evaluated the contribution of the different health indicators to the clustering separation using an embedded method for feature importance detection, namely, extra trees (settings: number of trees = 101, random state = 0) [12]. 

*(2) Estimation of Routines*. At the behavior layer of the DLM, we discovered behavioral routines by applying eigendecomposition at individual and group level as described in Section 2.4. The diet and activity spaces are considered separately. We estimated the minimum number of primary eigenbehaviors that was sufficient to describe individuals and group behavior variability with up to 90% of reconstruction accuracy. We repeated the same analysis considering the overall population to verify if the minimum number of primary eigenbehaviors was balanced across the groups. 

*(3) Prediction of Daily Behaviors Using a Portion of Data from the Same Day*. We tested the ability of eigendecomposition to predict diet and activity of a portion of a day by using the information of the preceding periods in the same day. For example, in the diet behavioral space, for a given subject, first the eigenbehaviors *u* are computed across all days (training days) except one (test day). The behavior in the test day (*T*) was then reconstructed as the linear combination of the eigenbehaviors of *M* elements or classes, *T* = ∑_*i*=1_^*M*^*ω*_*i*_*u*_*i*_. By applying least square fitting using only the classes of the first half of the test day (values of classes in P0, P1, and P2) it was possible to estimate the weights *ω*_*i*_ which together with the eigenbehaviors were used to estimate the behavior in the remaining period of the day (values of classes in P3, P4, and P5). We used leave-one-day cross-validation and we reported mean accuracy of reconstruction across subjects to evaluate the predictive capability of such method. The same approach was used for activity data, in which case values of classes in P0 and P1 (activities in the morning and afternoon) were used to predict values of classes in P2 (activities in the evening). 

*(4) Estimation of Day by Day Similarities*. For each individual, we investigated the similarities between days per diet and activity space, by projecting each day into the individual's behavior space and computing the Euclidean distance as described in Section 2.4. Large distances correspond to low degrees of similarity and small distances to high degrees of similarity. For each day, *n*, we determined the most similar day to it and we annotated it as the day index *d*_*n*_. We then built day index vectors, *D* = [*d*_1_, *d*_2_, *d*_3_,…, *d*_*n*_] for diet and *A* = [*d*_1_, *d*_2_, *d*_3_,…, *d*_*n*_] for activity (e.g., if *D* = [2,1, 7,…], the value at index 1 of *D* indicates the most similar day to day 1, day 2, the value at index 2 of *D* indicates the most similar day to day 2, day 1, the value at index 3 of *D* indicates the most similar day to day 3, day 7, etc.). By computing the percentage of overlap between values of *D* and *A*, we evaluated if day similarities were kept across different behavior spaces. If this overlap was high for an individual, it meant that repetitive diet and activity behavior were intertwined. 

*(5) Estimation of Similarities between Individuals*. Similarities between individuals were computed by projecting the average individual's behavior into each group behavior space and computing the Euclidean distance as described in Section 2.4. This analysis allowed us to identify subjects close to each other within the same group or within different groups and for which behavioral category, either diet or activity. The distances between individuals were then visualized using a gamified illustration. A dartboard-like figure is used which has the reference subject at the center and the other subjects positioned at different orbits with the proximity proportional to their similarity to the reference subject. 

*(6) Estimation of an Individual's Distance from a Cluster*. For each individual, we computed the Euclidean distances between the mean-adjusted behavior of the individual and their projection onto the group's behavior spaces, as described in Section 2.4. We used these behavior distances to predict group membership by applying different machine learning techniques, such as decision tree, random forest (settings: number of trees = 101), and support vector machine (settings: rbf kernel). Leave-one-subject-out cross-validation was used to evaluate the classification accuracy. Random forest and SVM hyperparameters were selected using the grid search method with cross-validation within the training set. This evaluation had the objective to determine if the clustering based on health indicators was also reflected at a behavioral level, in particular to identify for which behavioral space this statement was plausible. 

*(7) Evaluation of Health Indicators-Based Clustering*. As a final evaluation of our grouping method, we compared the average of individual distances from each group behavior, as obtained by the proposed unsupervised clustering and as obtained by random clustering. We first created random groupings by shuffling the individuals in the population 100 times, each time creating different grouping configurations, while keeping the number of groups and the number of the individuals within a group equal to the one obtained by unsupervised clustering. Then at each shuffle, the individual's Euclidean distances from his/her own group and opposite groups were computed and averaged; for example, in the case of two groups, group 0 and group 1, the means of within-group distances, *d*^00^ and *d*^11^, and the means of between group distances, *d*^01^ and *d*^10^, were obtained (e.g., *d*^00^ indicates mean distances of individuals of group 0 from the average behavior of group 0 when projected in group 0; *d*^01^ indicates mean distances of individuals of group 0 from the average behavior of group 1 when projected in group 1). This validation had a twofold objective: evaluating if our results could be obtained by chance and revealing the relationship between health indicators and behaviors.

The results in the following section are presented using the sequence of steps as defined above.

## 3. Results

### 3.1. Clustering of Population by Bodily, Physiological, and Metabolic Characteristics

**Figure 4:**
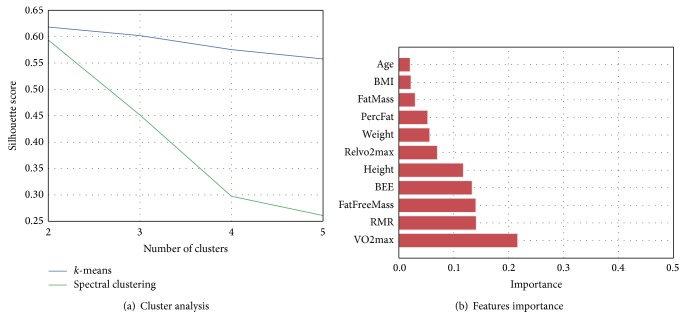
(a) Silhouette scores against number of clusters for* k*-means and spectral clustering. (b) Feature importance.

Unsupervised clustering applied to the health indicators listed in Table 1 revealed that, for both clustering methods,* k*-means and spectral clustering, the best clustering configuration was obtained when the population was separated into two groups; for a higher number of clusters, separation was poor and not representative (see silhouette score profile in Figure 4(a)).* K*-means showed slightly better separation than spectral clustering. Thus* K*-means was selected as the final clustering technique. The evaluation of feature importance obtained by applying extra trees classification showed that fitness level (VO2max) and basal energy expenditure (BEE) were the health indicators that mostly contributed to the clusters separation (see Figure 4(b)). Additionally, when inspecting the gender of the participants in the two groups we found that 92.3% of participants in group 0 were female and 87.5% of group 1 were male. This observation can be explained by known gender differences in fitness level, metabolism, and body composition (i.e., males are in general relatively fitter, taller, and heavier than female).

### 3.2. Estimation of Primary Eigenbehaviors

Primary eigenbehaviors were computed for each individual and for each group as explained in Section 2.5.

**Figure 5:**
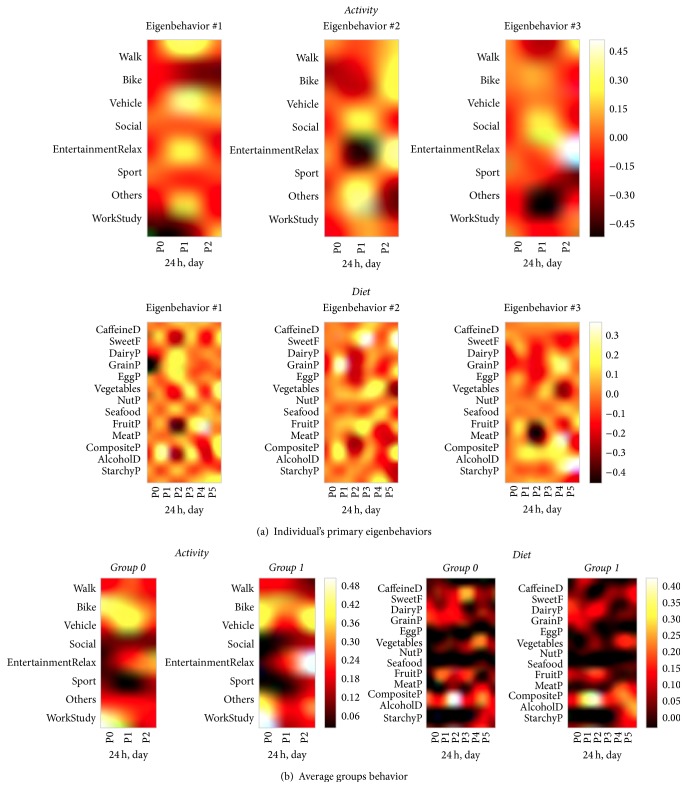
(a) Three primary eigenbehaviors for an individual belonging to group 0. (b) Average groups behavior.

In Figure 5(a), we show an example of the first three primary eigenbehaviors for a subject belonging to group 0 for the activity and the diet behavior spaces. For the activity space, the first eigenbehavior is representative of a routine in which the subject does activities other than work or study and he/she entertains or relaxes, walks, and uses transport systems during the middle of the day. The second eigenbehavior is representative of daily routines consisting of relaxation during the evening, other, and social activities during the day and evening cycling. The third eigenbehavior is similar but does not include other activities and has more evening walks. For the diet space, the first eigenbehavior is representative of a dietary routine for which grains are consumed during breakfast (P0) and sweets, fruit, and composite food are consumed during lunch time (P2). The second eigenbehavior includes dairy and egg products and composite food during lunch time, meat, vegetable, and starchy product intake during dinner (P4) and evening snacks (P5). The third eigenbehavior emphasizes the use of meat and grain products and alcohol use in the evening.

In Figure 5(b), average group behaviors are reported. In the activity space, both groups exhibit work and study activities in the morning. Individuals in group 0 cycle during the morning and use other transport systems during the day, while individuals in group 1 mostly cycle during the day and in the evening. Additionally they do more relaxing/entertainment activities during the evening. In the diet space, in both groups, individuals consume composite food during lunch time and at dinner. Group 0 has higher consumption of vegetables, fruits, and sweet, while group 1 consumes more alcohol at the end of the day. Visual inspection shows that the differences between average group behaviors were more predominant in activity than in diet space.

**Figure 6:**
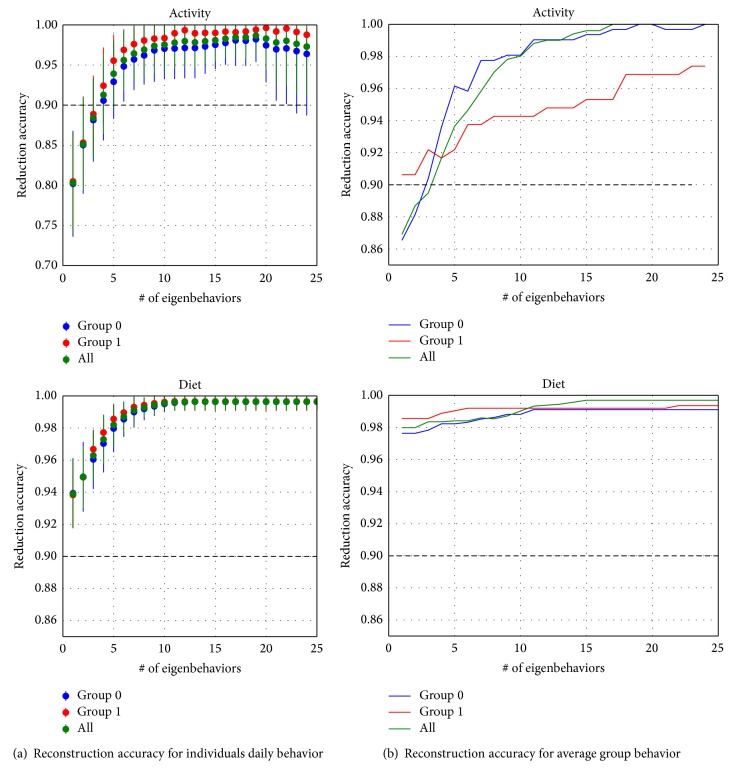
(a) Mean and standard deviation of daily reconstruction accuracy across days and individuals against the number of eigenbehaviors required for such reconstruction. (b) Mean reconstruction accuracy of group behaviors.

For both individuals and groups and in both diet and activity spaces, behaviors' reconstruction accuracy above 90% obtained with linear combination of eigenbehaviors could be reached using the first five to ten eigenbehaviors. Interestingly, dietary behavior required less number of eigenbehaviors than activity behavior at parity of accuracy of reconstruction (see Figure 6).

### 3.3. Prediction of Daily Behaviors Using a Portion of Data from the Same Day

**Figure 7:**
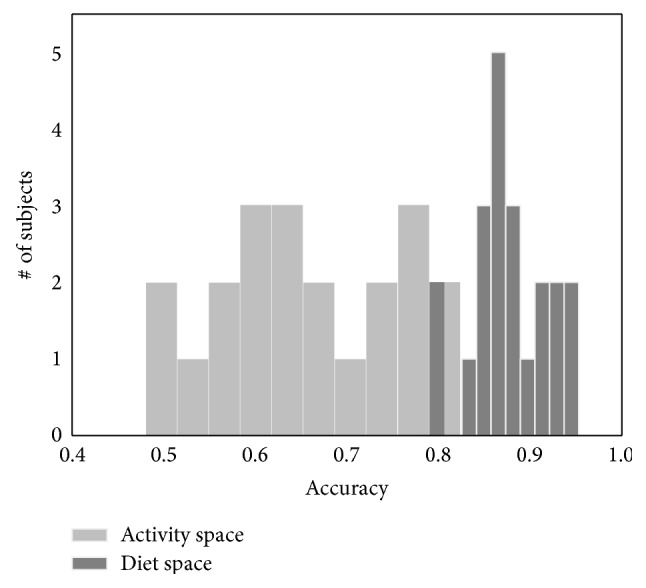
Prediction accuracy for the behavior during the last part of the day (P2, for activity; P3-P4-P5 per diet) given the behavior in the first part of the day (P0-P1 for activity, P0-P1-P2 for diet). An average of 66% accuracy is obtained for activity behavior, and 88% is obtained for diet behavior.

Results on the ability to predict an individual's behavior during a portion of a day are reported in Figure 7. Here, the distributions of prediction accuracy averaged across days for each subject are shown for the diet and the activity spaces. Mean prediction accuracy was higher for the diet behavior space (mean accuracy = 88%) than for the activity behavior space (mean accuracy = 66%).

### 3.4. Estimation of Day by Day Similarities

**Figure 8:**
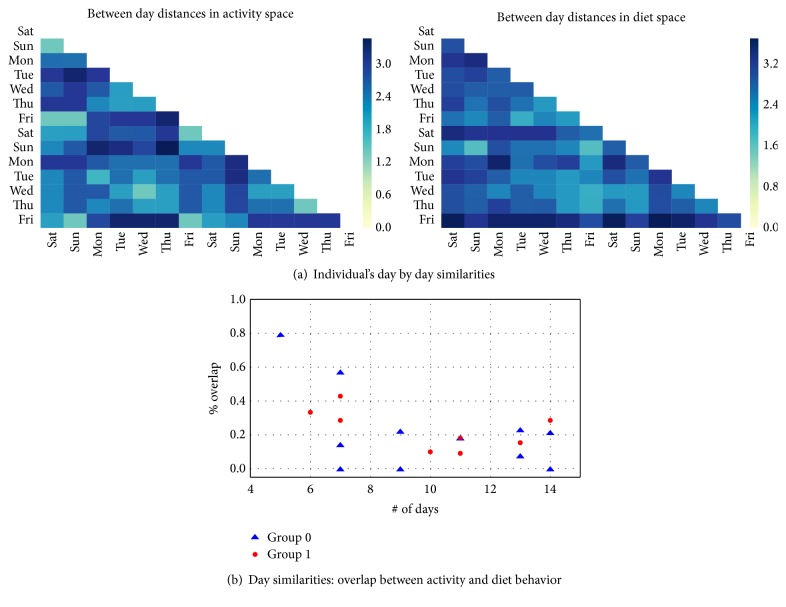
(a) Heatmap representation of between days Euclidean distances in activity and diet space for an individual belonging to group 0. (b) Percentage of overlap between diet and activity days day index vectors against number of annotated day. Each point corresponds to data from a different individual.

Day by day similarities were computed for each subject.  Figure 8(a) shows an example of Euclidean distances for an individual belonging to group 0, where each day is compared to the others separately for diet and activity spaces. For each day of a subject, the most similar days are considered to form the day index vector as explained in Section 2.5.  Figure 8(b) shows that the percentage of overlap between day index vectors in diet and in activity spaces decreased with the number of days. This was a trivial result because the number of days corresponds to the size of the day index vectors for a subject and the higher the dimension of the day index vector, the higher the chances to find differences between days. No statistical differences (*p value < 0.05*) were found when comparing the percentage of overlap in individuals between the two groups. These observations showed that over a period of up to 14 days, for our study population, there was a chance of about 20% that if two days were similar in activity space, they were also similar in the diet space, independently of the group belonging.

### 3.5. Estimation of Similarities between Individuals

Similarities between individuals were computed by projecting individuals in different groups and calculating the Euclidean distances between individual pairs.

**Figure 9:**
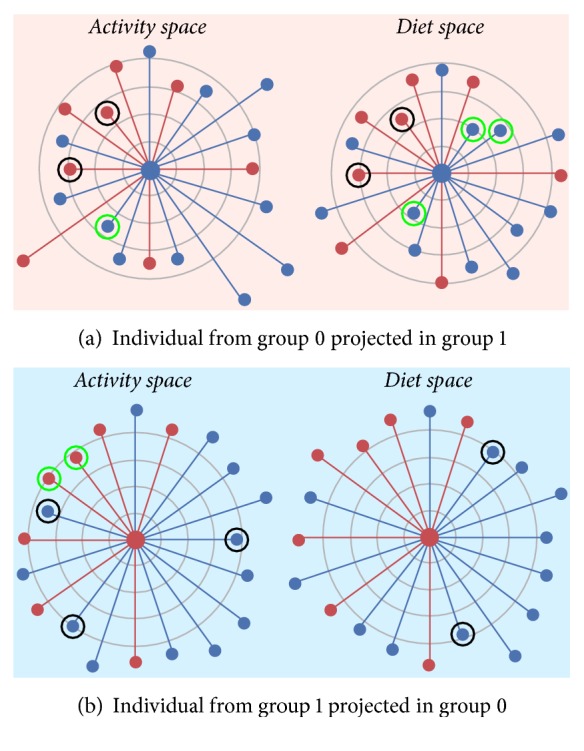
Gamified illustration of distances between individuals. Individuals (a) and (b) are in the center of the dartboards. (a) is projected in group 1 (indicated by the red background); (b) is projected in group 0 (indicated by the blue background). Individuals belonging to group 1 and group 0 are represented by red and blue dots, respectively. Equally spaced rays represent distances of different subjects from the central subject ((a) or (b)). Gray concentric rings are equally spaced reference distances to facilitate distance perception (in each dartboard the distances between rings are the same). Green and black circles highlight the closest individuals, belonging to the same group or the other group, respectively.

We represented such distances for each subject using a dartboard-like representation as shown in Figure 9, with the subject in the middle of the board and the distance from other subjects being represented by different rays at equally spaced angles. Concentric rings with equally spaced ray were also drawn to provide reference distances from the center. The left two dartboards represent an individual (a) from group 0 (blue points) projected into group 1 (red background) in the activity and diet space; the right two dartboards represent an individual (b) from group 1 (red points) projected into group 0 (blue background) in the activity and diet space. When projected into group 1, individual (a) shows activity behavior similar to two people from group 1 (see black circles in the nearest occupied concentric rings) with whom he/she shares also similar diet behavior, meaning that individual (a) shared similar behaviors with same people in different spaces. His/her diet behavior was in general similar to the people belonging to her/his group (see green circles in the nearest occupied concentric rings). When projected in group 0, individual (b) shows diet behavior more similar to individuals of group 0 than to the group in which he/she belonged (see how blue points are closer to the center than red points, and also notice more black circles than green circles in the nearest occupied concentric rings). In this case the most similar individuals to (b) with respect to diet and activity were not the same, meaning that individual (b) shared similar behaviors with different people in different spaces.

### 3.6. Estimation of an Individual's Distance from a Cluster

The Euclidean distances between the mean-adjusted behavior of the individual and its projection onto the group's behavior for diet and activity spaces were computed as explained in Section 2.5.

**Figure 10:**
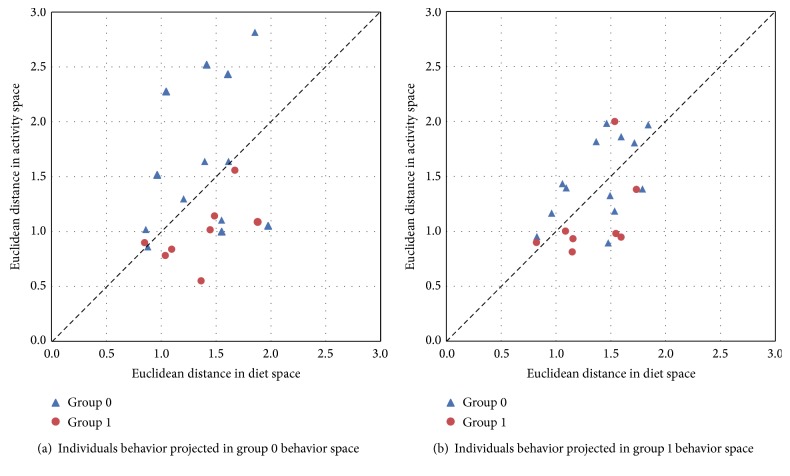
The cross-validated distance between individuals and the activity and diet behavior spaces of group 0 (a) and of group 1 (b). Dotted black line is the identity line used as reference for visual inspection.

Figure 10 shows distances of individuals from group 0 (Figure 10(a)) and from group 1 (Figure 10(b)), where each axis corresponds to a different behavior space. Group separation was mostly driven by activity behavior rather than by diet behavior, independently of the group onto which the subject was projected. In particular, individuals from group 0 had more variability in activity behavior, while individuals from group 1 had more regular activity behavior. Dietary behavior varied similarly across groups. If the diet and activity spaces were linearly correlated and if differences between the average group behaviors were comparable across diet and activity, subjects belonging to group 0 would have exhibited lower distance both in diet and activity when projected onto the group 0 behavior space (blue triangles, in Figure 10(a)). Similarly subjects belonging to group 1 would have exhibited lower distance both in diet and activity when projected onto group 1 behavior space (red points, in Figure 10(b)). Despite these observations, we tested the use of these behavioral distances for detecting an individual's group membership. For all the used machine learning techniques the classification accuracy was below 70% (64% for decision tree, 60% for random forest, and 48% for support vector machine).

### 3.7. Evaluation of Health Indicators-Based Clustering

The proposed health indicators-based clustering was compared with random clustering as explained in Section 2.5.

**Figure 11:**
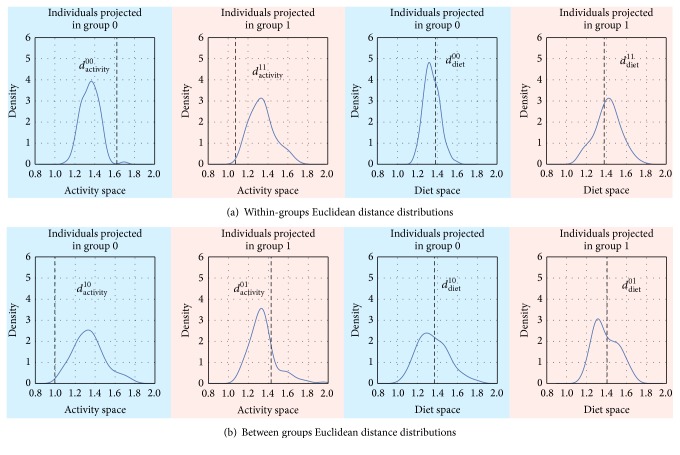
Distribution of mean distances of individual's behavior from own group behavior (a) and from the other group behavior (b) as obtained by shuffling individuals across groups. Background colors represent in which group individuals are projected, previous distance computation (red for group 1 and blue for group 0). Dashed lines refer to values of distances obtained by the proposed unsupervised clustering.

Figure 11(a) shows within-group distance distributions, where *d*_activity_^00^ and *d*_diet_^00^ represent mean distances across individuals of group 0 from the behavior of group 0, when each individual is projected in the activity and diet spaces of group 0. *d*_activity_^11^ and *d*_diet_^11^ represent mean distances across individuals of group 1 from the behavior of group 1, when each individual is projected in the activity and diet spaces of group 1. In Figure 11(b), between group distance distributions are shown, where *d*_activity_^01^ and *d*_diet_^01^ represent mean distances across individuals of group 0 from the behavior of group 1, when projected in the activity and diet spaces of group 1. *d*_activity_^10^ and *d*_diet_^10^ represent mean distances across individuals of group 1 from the behavior of group 0, when projected in the activity and diet spaces of group 0. In each plot the dashed lines correspond to the mean distances as obtained by our clustering. Health indicators-based clustering produced same outcomes of random clustering for distances computed in the diet space independently of the group projection, and this is indicated by the dashed lines lying in the bulk of the distances distributions. On the other hand, health indicators-based clustering was different from random clustering for distances computed in the activity space. In this case the dashed lines lay outside the bulk of the distances distributions. In particular, small *d*_activity_^11^ indicated that individuals in group 1 have very similar within-group activity behaviors, large *d*_activity_^00^ indicated that individuals in group 0 had very different within-group activity behaviors, small *d*_activity_^10^ indicated that individual in group 1 had also very similar between-group activity behavior, and large *d*_activity_^01^ indicated that individual in group 0 had also very different between-group activity behavior.

## 4. Related Works and Discussion

The increasing availability of data from web, mobile, and wearable sensing in combination with the increasing usage of machine learning techniques is facilitating the employment of holistic approaches in the design of lifestyle applications, such as recommender systems for behavior change [13]. In particular, the possibility to aggregate several data types, such as diet, activity, social interactions, and physiological responses to physical and mental stress, is contributing to improving our understanding of the relationships between behavioral and physiological dynamics and health outcomes. Nevertheless, very few studies have been oriented to modeling and predicting behavior dynamics for lifestyle applications. Prediction of behaviors was explored in the context of social network in [5] using mobile technology and in [14] using wearable cameras. Activity prediction models were also proposed in [4] using infrared motion sensors installed in resident smart home apartments and in [15, 16] using social media data from Facebook, Renren, and Twitter. In [17] a multiscale adaptive personalized model that quantifies the effect of both lag and behavior cycle for predicting future behavior was presented. In none of these studies, correlations between behavior and health indicators were considered. In this work we proposed a novel approach, the double layer methodology, to model and uncover such dependencies. We demonstrate the use of the DLM to the problem of tracking lifestyle from self-reports of diet and activity behaviors. In the first layer of the DLM, unsupervised machine learning techniques were used to clusters individuals on the basis of health indicators. In the second layer of the DLM eigendecomposition techniques were used to model individual and group behaviors. Eigendecomposition was selected because compared to other techniques such as Markov models, they had the advantage of incorporating temporal patterns across different timescales [14].

The computational steps at the first layer of the DLM revealed that our population could be clustered in two groups and that rest metabolic rate and fitness level could be considered relevant health indicators for clustering our study population. Resting metabolic rate is linearly related to the basal energy expenditure and depends on factors such as gender, age, weight, and height [11]. Fitness level is also related to gender, age, and muscular mass and it is recognized as quantitative predictor of all-cause mortality and cardiovascular events in healthy men and women [10]. They are both gender dependent, which means that clustering based on these health indicators captured also gender differences. It is important to state that unsupervised clustering does not label individuals in predefined categories, for example, healthy or not healthy, and this allows overcoming problems related to ethical considerations. Instead it allows finding the hidden patterns in a population and to group individuals on the basis of bodily, physiological, and metabolic characteristics that can be considered as a proxy of health status.

At the second layer of the DLM, the eigendecomposition technique was applied for detecting behavioral routines and a validation of the health indicators-based clustering technique was proposed. We showed that, for our population, dietary routines were more regular than activity routines, both at individual and at group level. At parity of reconstruction accuracy, the number of primary eigenbehaviors needed for reconstructing diet behavior was minor than for activity behavior. This outcome was also reflected in the higher accuracy of prediction of diet behaviors in portion of a day (88%), compared to prediction of activity behaviors (66%). These results should be considered in the light of the assumption upon which eigenbehavior analysis is based: eigenvectors with large eigenvalues contain most of the data variability. It follows that small number of primary eigenbehaviors corresponds to higher regularity of a routine. This assumption might not be necessarily true for routines across different behavioral spaces, and it can lead to incorrect interpretation if the data are noisy and sparse.

When computing between days similarities we found that the overlap between diet and activity routines was dependent on the length of the observation period and that, over a period of 14 days, there was a chance of about 20%, on average that if two days were similar in the activity space, they were also similar in the diet space. In a real scenario, overlap of routines across different behavioral spaces can occur, for example, if an individual is undergoing a physical training for which daily consistency of exercise and diet timing are required. Identification of day similarities can be used to estimate the frequency of a routine, especially for datasets covering longer recording period. In the context of recommendation, it could allow inferring in which days the user is more prone and open to receive feedback for behavior change.

When distances of individual behaviors from the average group behavior in diet and activity spaces were used to detect the individual's group belonging, a classification accuracy below 70% was obtained. This result reflected the nonlinearity between health indicators and behaviors, meaning that the people having the same range of health indicators did not necessarily exhibit similar behaviors or that people with different health indicators could have exhibited similar behaviors, across different behavior spaces.

Finally, the comparison of the proposed health indicators-based clustering techniques against random clustering revealed that the results obtained in the diet space could have been produced by chance. This consideration also extends to the results on the regularity of diet behavior, particularly, because diet data were noisier and sparser (as shown in the example of behavior matrix in the diet space in Figure 1(b)). On the other end, this validation analysis also revealed that the grouping based on health indicators was actually mapped at a behavior level in the activity space. In this space, our clustering produced different results from random clustering, also showing different behavior patterns across different groups. For example, individuals from group 1 had activity behaviors close to average behavior of both groups and individuals from group 0 had activity behaviors distant from the average behavior of both groups. In the ideal case of tight and straightforward relationships between health indicators and behaviors, we would expect the within-group behavior distances obtained to be minimal and the between group behavior distances to be maximal for both activity and diet spaces. In reality, since individual bodily characteristics might only partially contribute to one's behavior, such relationship should be discovered only for some groups in specific behavior spaces, in our case for group 1 and activity space.

Taken together, our results show the limits of applying eigendecomposition to model behavioral routines. In particular its sensitivity to noise and its assumption of linearity should be carefully accounted for, considering the sparse and complex nature of human behavior. The proposed DLM and validation technique allowed identifying the limitations of eigendecomposition and finding behavioral correlates of health indicators for our study population.

We believe that the DLM, here demonstrated as a tool for automatic analysis of behaviors from self-reports, has a potential use for the development of tailored recommender systems that account for differences in behaviors across individuals in different groups. In particular, it could be applied to characterize specific health profiles. Also, the proposed dartboard-like visualization could be used to facilitate the selection of advices to be given to an individual, not only on the basis of the collective behavior of the group to which he/she belongs, but also on the basis of his/her similarities to individuals belonging to other groups [18]. The dartboard-like visualization and the insights produced by the DLM could increase the richness of recommendations to an individual to stimulate behavior change and could also be used to show the hypothetical consequences of an unhealthy behavior to a healthy user, for example, by showing the profile of an unhealthy population exhibiting similar behaviors.

The major limitations of our study lie in the typology of our data source (semantic information from self-report diary) and in the size of our dataset (in terms of both number of participants and number of observations per subject). Self-reporting of diet and activity, in the form of diary or 24-hour recall questionnaires, is largely employed in large epidemiological studies, representing a trivial and at hand solution to collect information on individual behaviors. However, self-reporting measures require considerable efforts from the observant, and lack of motivation, memory decay, and imprecision of recall can compromise the reliability of such information for accurate behavior tracking [19, 20]. Imprecision of self-reporting in our data might have produced misleading results in the projection of the individual behavior in a specific behavioral space or group or in the computation of between-subject differences and within-subject day by day similarities. Self-reported diaries should therefore be juxtaposed or complemented with more quantitative and objective readings, such as those provided by personal tracking devices for continuous monitoring of activity and physiology (i.e., smartphones and wearable health monitors). Finally, the results presented in this study model the behaviors of a limited and specific healthy young population, monitored for a short period of time. With the use of cross-validation and comparison to randomly shuffled data we could prove the solidity of our results. At the same time, large-scale studies with wider population varying per demographics, ethnicity, and socioeconomic status, and longitudinal studies which are able to account for the routines that activate across short and long time scale [21, 22] should be considered to prove the validity of our approach as a supporting tool for behavior change applications (i.e., weight management, smoking cessation).

## 5. Conclusion

Self-report and daily diary both in the form of manual annotation and mobile applications constitute up to today the most commonly used and intuitive method for recording behavior routines either for personal self-tracking or for clinical prescription. In this work, we propose the DLM for analyzing lifestyle patterns emerging from self-reports of daily diet and activity. Despite the limited size of our dataset, we show the potential of the DLM for the identification of behavior routines and of their relationship with health indicators. We evaluated the limits of eigenbehavior decomposition when applied to behavior data for lifestyle applications. We also proposed a novel gamified representation of individual and group behaviors which could be used to support selection of advices in personalized recommender systems for behavior change.

The novelty of the DLM lies in the criteria that were used for grouping individuals by health indicator surrogates. We showed that such separation partially reflects in group behaviors, for particular behavior spaces, which indicates that health condition and intrinsic physical characteristics can play a role in the exhibition of a particular behavior. Although at this stage, causal relationships between behavior and health status cannot be proven, this result has potential for the application in behavioral therapy for improving a person lifestyle and health.

Future extensions of the DLM will include the use of other sources of information, such as data from wearables and ubiquitous sensing and the validation of other behavior tracking algorithms for overcoming the limitations of eigenbehavior decomposition. In particular, alternative definitions of routines will be considered (e.g., the definition of de Lira et al. (2014) [23] of semantic regularity profiles) (where entropy is used as a measure of spatial and temporal regularity of a behavior) and alternative algorithms to models behavior dynamics and long-range temporal correlations such as autoregressive models and neural networks [17, 24, 25] will be evaluated.

## Figures and Tables

**Table 1:** Study population demographics and health indicators.

Variable	Unit	Mean ± standard deviation
Age	Years	26 ± 6
BMI	kg/m^2^	22.7 ± 2.5
Weight	kg	69.7 ± 10.8
Height	cm	174.9 ± 9.2
VO2max	ml/min	3009 ± 679
Relative VO2max	ml/kg/min	43 ± 6.8
RMR	kcal/min	1.2 ± 0.16
BEE	kcal/day	1567 ± 215
Fat mass	kg	14.4 ± 6.9
Fat free mass	kg	55.3 ± 9.4
Percentage of fat	%	20.4 ± 7.7

**Table 2:** Activity and diet classes.

	Examples of words included in the class
Activity classes	
Entertainment/relax	Shop, travel, watch, game, play, computer, TV, movie
Work/study	Exam, homework, read, work, lesson, university, lecture, school, study
Sport	Run, sport, gym, hockey, swim, fitness, soccer, workout
Social	Meet, friends, call, party, talk, phone, parent, visit
Vehicle	Car, bus, train, taxi, drive
None	—
Others	Wait, household, pack, shower
Walk	Walk
Bike	Bike, cycle
Diet classes	
Fruit product	Fruit, orange, apple, banana, kiwi, sultana, pineapple, smoothie, juice
Grain product	Noodles, oatmeal, muesli, bread, macaroni
Composite product	Sandwich, pizza, soup, rice, pasta, lasagna, hamburger
Vegetables	Cucumber, spinach, carrot, pumpkin, broccoli, tomato
Meat product	Beef, bacon, meat, sausage, chicken, steak
Snacks	Nut, pie, candy, ice cream, chocolate, cake, snack, cookie
Alcohol drink	Beer, wine, alcohol
Others	Butter
Seafood	Fish, tuna, salmon
Caffeine drink	Cola, tea, coffee, cappuccino
Starchy product	Potato, chip, fries
Dairy product	Shake, milk, cheese, yoghurt

## References

[1] Norman G. J., Zabinski M. F., Adams M. A., Rosenberg D. E., Yaroch A. L., Atienza A. A. (2007). A review of eHealth interventions for physical activity and dietary behavior change. *American Journal of Preventive Medicine*.

[2] Ravi N., Dandekar N., Mysore P., Littman M. L. Activity recognition from accelerometer data.

[3] Pantic M., Rothkrantz L. Ü. M. (2000). Automatic analysis of facial expressions: the state of the art. *IEEE Transactions on Pattern Analysis and Machine Intelligence*.

[4] Nazerfard E., Cook D. J. Using bayesian networks for daily activity prediction.

[5] Eagle N., Pentland A. S. (2009). Eigenbehaviors: identifying structure in routine. *Behavioral Ecology and Sociobiology*.

[6] Altini M., Casale P., Penders J., Amft O. (2016). Cardiorespiratory fitness estimation in free-living using wearable sensors. *Artificial Intelligence in Medicine*.

[7] Eagle N., Pentland A. S. (2006). Reality mining: sensing complex social systems. *Personal and Ubiquitous Computing*.

[8] Popat S. K., Emmanuel M. (2014). Review and comparative study of clustering techniques. *International Journal of Computer Science and Information Technologies*.

[9] Huber M., Knottnerus J. A., Green L. (2011). How should we define health?. *British Medical Journal*.

[10] Kodama S., Saito K., Tanaka S. (2009). Cardiorespiratory fitness as a quantitative predictor of all-cause mortality and cardiovascular events in healthy men and women: a meta-analysis. *The Journal of the American Medical Association*.

[11] Stiegler P., Cunliffe A. (2006). The role of diet and exercise for the maintenance of fat-free mass and resting metabolic rate during weight loss. *Sports Medicine*.

[12] Geurts P., Ernst D., Wehenkel L. (2006). Extremely randomized trees. *Machine Learning*.

[13] Jager W. (2003). Breaking bad habits: a dynamical perspective on habit formation and change. *Human Decision-Making and Environmental Perception—Understanding and Assisting Human Decision-Making in Real Life Settings. Libor Amicorum for Charles Vlek*.

[14] Clarkson B. P. (2002). *Life patterns: structure from wearable sensors [Ph.D. thesis]*.

[15] Radinsky K., Svore K., Dumais S., Teevan J., Bocharov A., Horvitz E. Modeling and predicting behavioral dynamics on the web.

[16] Zhu Y., Zhong E., Pan S. J., Wang X., Zhou M., Yang Q. Predicting user activity level in social networks.

[17] Preum S. M., Stankovic J. A., Qi Y. MAPer: a multi-scale adaptive personalized model for temporal human behavior prediction.

[18] Elahi M., Ricci F., Rubens N. (2016). A survey of active learning in collaborative filtering recommender systems. *Computer Science Review*.

[19] Martin L. J., Su W., Jones P. J., Lockwood G. A., Tritchler D. L., Boyd N. F. (1996). Comparison of energy intakes determined by food records and doubly labeled water in women participating in a dietary-intervention trial. *The American Journal of Clinical Nutrition*.

[20] Sallis J. F., Saelens B. E. (2000). Assessment of physical activity by self-report: status, limitations, and future directions. *Research Quarterly for Exercise and Sport*.

[21] Aarts H., Paulussen T., Schaalma H. (1997). Physical exercise habit: on the conceptualization and formation of habitual health behaviours. *Health Education Research*.

[22] Lally P., Van Jaarsveld C. H. M., Potts H. W. W., Wardle J. (2010). How are habits formed: modelling habit formation in the real world. *European Journal of Social Psychology*.

[23] de Lira V. M., Rinzivillo S., Renso C., Times V. C., Tedesco P. C. Investigating semantic regularity of human mobility lifestyle.

[24] Devooght R., Bersini H. Collaborative filtering with recurrent neural networks. https://arxiv.org/abs/1608.07400.

[25] Hawkins J., George D., Niemasik J. (2009). Sequence memory for prediction, inference and behaviour. *Philosophical Transactions of the Royal Society of London B: Biological Sciences*.

